# An Extraordinary Advertisement

**Published:** 1889-11

**Authors:** 


### An Extraordinary Advertisement.

The following advertisement appeared in a recent issue of the *Herald*:—"Robbery.—Any person having seen any one meddling with the artificial teeth in this window during the hours between ten o'clock Saturday night, July 13, and Sunday up to four o'clock in the afternoon, and can give any description of the party so acting, will kindly call on me at once, will receive a reward of £20." Here follow the name and address of a well-known dentist. The practical joke that gave rise to this advertisement is said to have been making the sets of teeth into rat traps, with a piece of cheese placed by way of bait.

As a readily prepared antidote for acute arsenical poisoning, Prof. Holland gives the following:

| | | | | |
|---|---|---|---|---|
| ℞. | Liquor. ferri tersulphat , | | | |
| | Aquæ destillat | aa. | f℥ij. | M. |
| ℞. | Magnesiæ | | ʒiiss. | |
| | Aquæ destillat | | f℥viij. | M. |

Sig.—Mix the two solutions and give a tablespoonful, diluted, every five minutes, as required.—*Canada Med. Record.*

The John Hopkins Hospital of Baltimore was formally opened May 7. It construction was begun more than ten years ago, and the cost has been over $2,000,000. This institution is one of the finest and most complete in the world, embracing seventeen buildings and covering four acres of ground, surrounded by ten acres more, which is laid out in a beautiful park.

